# POMDP-Based Real-Time Path Planning for Manipulation of Multiple Microparticles via Optoelectronic Tweezers

**DOI:** 10.34133/2022/9890607

**Published:** 2022-11-02

**Authors:** Jiaxin Liu, Huaping Wang, Menghua Liu, Ran Zhao, Yanfeng Zhao, Tao Sun, Qing Shi

**Affiliations:** ^1^Intelligent Robotics Institute, School of Mechatronical Engineering, Beijing Institute of Technology, Beijing 100081, China; ^2^Key Laboratory of Biomimetic Robots and Systems (Beijing Institute of Technology), Ministry of Education, Beijing 100081, China; ^3^School of Medical Technology, Beijing Institute of Technology, Beijing 100081, China; ^4^Beijing Advanced Innovation Center for Intelligent Robots and Systems, Beijing Institute of Technology, Beijing 100081, China

## Abstract

With high throughput and high flexibility, optoelectronic tweezers (OETs) hold huge potential for massively parallel micromanipulation. However, the trajectory of the virtual electrode has been planned in advance in most synchronous manipulations for multiple targets based on an optically induced dielectrophoresis (ODEP) mechanism, which is insufficient to ensure the stability and efficiency in an environment with potential collision risk. In this paper, a synchronously discretized manipulation method based on a centralized and decoupled path planner is proposed for transporting microparticles of different types with an OET platform. An approach based on the Kuhn-Munkres algorithm is utilized to achieve the goal assignment between target microparticles and goal positions. With the assistance of a visual feedback module, a path planning approach based on the POMDP algorithm dynamically determines the motion strategies of the particle movement to avoid potential collisions. The geometrical parameters of the virtual electrodes are optimized for different types of particles with the goal of maximum transport speed. The experiments of micropatterning with different morphologies and transporting multiple microparticles (e.g., polystyrene microspheres and 3T3 cells) to goal positions are performed. These results demonstrate that the proposed manipulation method based on optoelectronic tweezers is effective for multicell transport and promises to be used in biomedical manipulation tasks with high flexibility and efficiency.

## 1. Introduction

The manipulation of cells and cellular objects is one of the most promising methods applied in biomedical researches such as drug delivery [1], tissue engineering [2], personalized diagnosis, and treatment [3]. As a typical cell manipulation methodology, cell transport represents a series of significant tasks in biomedical researches, such as cell isolation [4], cell patterning [5], and cell assembly [6]. For example, to construct a drug test model or clinical alternatives with physiological properties more similar to liver hepatic lobules, it is incredibly beneficial to transport parenchymal cells and nonparenchymal cells and arrange them alternately regionalized with an outline of a six-sided cylinder and radial distribution. The reason is that the arrangement pattern of cells has great effects on the status of individual cells and functional expression of resultant tissues through direct contact with adjacent cells or exchange of soluble factors. Therefore, transporting cells to respective designated locations is also the preliminary steps in the preparation for many subsequent cellular experiments (e.g., optimization of culture conditions [7] and studies of cell-cell interactions [8]). Through applying theories and methodologies in robotics to biological micromanipulation tasks, robotic cell transport techniques have constantly developed to be more versatile and efficient [9], spanning from contact actuated methods (including microprobe [10], micropipette [11], microgrippers [12], and atomic force microscope cantilevers [13]) to wireless actuated methods (including magnetic tweezers [14, 15], acoustic tweezers [16], optical tweezers [17], etc.) However, the independent and parallel motion control of each target in cell clusters is quite challenging for most robotic cell transport techniques.

Among these techniques, optoelectronic tweezers (OETs), also known as optically induced dielectrophoresis (ODEP, first proposed in 2005 by Chiou and coworkers [18]), not only have considerable performance in cluster manipulation [19] but also enable differential control over each individual target [20], which holds huge potential for the massively parallel manipulation of cells [21]. In addition to its high-throughput characteristics [22] (being able to form up to 50,000 virtual electrodes simultaneously [18]), OETs merely require 1/500 of optical power to generate traps with stiffness similar to OTs [23], which demonstrates its high efficiency and noninvasive features for intricate cell manipulation tasks. Furthermore, the mechanism of ODEP has been extensively utilized for modular assembly [24], cell rotation [25], actuation and control of microrobot [26, 27], cell purification and characterization [28], etc., which are comprehensively discussed in [29, 30]. In recent years, researchers have attempted to utilize programmable schemes [19, 31–33] based on the ODEP mechanism to facilitate the efficiency and accuracy of micromanipulation. Furthermore, the visual tracking algorithm was applied to provide feedback information for automated manipulation actuated by OETs [31]. In the initial manipulation environment, cells and impurities are usually distributed in a random manner throughout the whole liquid chamber. Scattered impurities and randomness caused by Brownian motion are nonnegligible issues for the smooth and efficient transport of multiple cells. However, the trajectories of virtual electrodes are usually planned offline or semiplanned in advance or even just manually controlled. The real-time motion decisions for target microparticles at microscale based on the ODEP mechanism is basic and crucial in cell transport tasks.

To avoid collisions between each target cell and microobjects (other target cells and obstacles) [34], real-time path planning for multiagents is necessary for discretized manipulation of multiple cells. Traditional robot studies have developed a comprehensive account of path planning algorithms, including the A^∗^ algorithm [35], RRT algorithm [36], and particle swarm optimization algorithm [37]. The characteristics and main applications of these algorithms were discussed in detail [38]. In particular, the partially observable Markov Decision Process (POMDP) has significant advantages in imperfectly known and dynamic environments. The random Brownian motion and uncertainty of response for control makes transportation tasks in the microenvironment more complicated. In recent years, path planning algorithms have been increasingly applied to biological micromanipulation robot systems, mainly to optical tweezer platforms. For example, Ju et al. demonstrated a dynamic path planner based on the RRT algorithm to address the randomness of the liquid environment produced by Brownian motion [39]. Despite significant breakthroughs in multiparticle path planning based on optical tweezers, synchronously discretized manipulation of different types of microparticles (e.g., biological cells) remains challenging. Moreover, limited by the maximum number of laser traps generated by the optical tweezer platform, the number of microparticles that can be transported simultaneously is not satisfactory enough. Considering OET's characteristics of flexibility and high throughput, a discretized manipulation method of multitype microparticles based on optoelectronic tweezers is of significant value in biomedical studies.

In this study, a decoupled POMDP-based path planning method for synchronously discretized transport of multiple microparticles with an OET manipulation platform is proposed. To actuate different types of microparticles synchronously, the virtual electrode is optimized into an appropriate size for each type of microparticle through numerical simulation of DEP force. To deal with the potential collisions caused by the movement of obstacles, a visual feedback module was integrated into the path planner, participating in every step of the strategy. Experimental results demonstrated considerable performance of the proposed discretized manipulation method in the OET platform and also indicated the feasibility of applying the mentioned methods to biological micropatterning. Furthermore, the future prospects and challenges of applying the proposed method in more intricate biomedical studies are discussed.

## 2. Materials and Methods

### 2.1. Experimental Design

**Figure 1:**
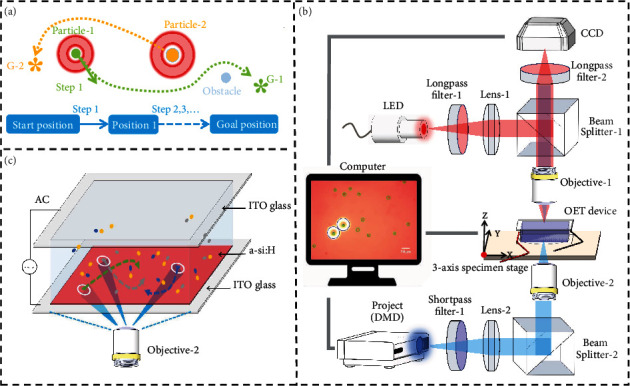
Conceptual overview of discretized manipulation of multiple microparticles based on OET manipulation platform. (a) Schematic of path planning for multiple particles by avoiding collisions. (b) Schematic of the manipulation platform based on optoelectronic tweezers. (c) 3D schematic of the ODEP chip.

To discretely manipulate multiple microparticles with an optoelectronic tweezer platform, the appropriate virtual electrode mode for each type of particles was investigated by simulation as a basis for experiments. Before transporting the particles, the goal position of each target particle was determined through the optimal goal assignment algorithm. Through visual feedback module participating in every strategy decision, all current environmental information and control statuses were detected as feedback information to prevent the risk of particles escaping from OET traps and accidental collisions. On this basis, microparticles of different types were smoothly transported and reached the goal position by avoiding collisions with obstacles and other target microparticles, as shown in Figure 1(a). Furthermore, experiments of micropatterns containing multiple particles were performed through the discretized manipulation method of multiple microparticles, which is beneficial for future biomedical studies.

The manipulations of the microparticles rely on the dielectrophoresis force provided by the OET platform (Figure 1(b)). The light pattern used to form the virtual electrode is generated by a digital projector and projected onto the ODEP chip through a condenser lens and an objective. A Light Emitting Diode (LED) applies a light source to illuminate the manipulation workspace. The combination of a long-pass filter and short-pass filter reduces the brightness of the light pattern in sight view, so that the environment and microparticles could also be clearly observed. A computer controls the movement of the ODEP chip for determining which area to manipulate by a 3-axis specimen stage. The real-time image offered by CCD is processed and analyzed by a computer, and then, the path planner formulates the next strategy based on the feedback information and executes it through the light pattern. The signal generator provides an AC voltage of up to 10 VPP for the ODEP chip as the basis for generating a nonuniform electric field.

The ODEP chip consists of three layers, as shown in Figure 1(c). The top layer is a piece of glass coated with an indium tin oxide (ITO) film on one side; the bottom layer is also a piece of ITO glass, but an additional film of photoconductive material (hydrogenated amorphous silicon) is covered. Double-adhesive tape connects the top and bottom electrode plates to form a chamber for holding the solution with microparticles. The photoconductor-coated substrates are illuminated with light to change the conductivity of the local area, so that a nonuniform electric field is generated. The time-average dielectrophoresis force exerted on a microparticle can be defined as

(1) FDEP=2πr3εmReKw∇E2.

### 2.2. Simulation Analysis

To analyze the behavior of microparticles in the optical-induced nonuniform electric field and optimize the geometric parameters of the virtual electrode, numerical simulation of the dielectrophoresis mechanism on microparticles was performed using the AC/DC module in COMSOL software. The 3D simulation model was also designed by COMSOL in order to parameterize the pivotal variables (width of virtual electrode, radius of OET trap). Based on the model, the electric field and electric potential were simulated as preparation for estimating the numerical value of the dielectrophoresis force. Since the size of the polystyrene bead is not much smaller than the dimension of electric field nonuniformity, the Maxwell stress tensor method is more suitable for approximating DEP force than the method of Equation (2), according to

(2) $$\begin{array}{c} F_{DEP}=\int_{S}T^{MST}\cdot ndS, \end{array}$$

where *S* represents the surface enclosing the microparticle, *n* is the unit vector perpendicular to *dS* and pointing to the inside of the microparticle, and *T*^*MST*^ is the Maxwell stress tensor of the electromagnetic field.

### 2.3. Path Planning Approach

**Figure 2:**
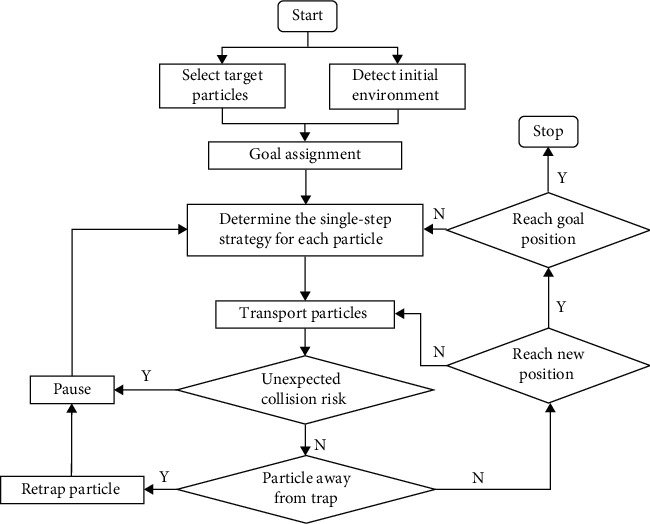
Flowchart of the discretized manipulation method.

The path planner dynamically controls the real-time positions and movement strategies of the microparticles and avoids collisions through global coordination to achieve discretized manipulation based on the optical-induced dielectrophoresis mechanism. The flowchart of the proposed manipulation method is shown in Figure 2. Target particles and goal positions could be manually selected by the operator through the human-computer interaction interface or are automatically determined by decision with reference to initial environmental information through visual feedback. The optimal goal assignment approach only worked once at the beginning of one manipulation task. By minimizing the total distance of particle movement, the goal assignment approach reduced the risk of collision and particle detachment from control and improved the efficiency of multitarget discretized manipulations. At the beginning of each control period, the path planner formulated a single-step optimal strategy for every target microparticle based on the location information of the obstacles recognized by the visual feedback module. Target microparticles were transported to the temporary target position at a preset speed according to the strategy. In the meantime, the location of the target microparticle and environmental information were detected in real time. Once there is a potential collision, the manipulation is suspended and the movement strategy is redetermined. If the microparticle escapes the light pattern, its new position is detected, and then, the microparticle will be retrapped by the virtual electrode. The current control period ended when the microparticles reached temporary target positions. The execution and decision-making of particle movement were repeated in this way until the manipulation task was completed.

#### 2.3.1. Goal Assignment

For multitarget manipulation tasks, it is of great significance to establish the optimal goal assignment between the goal positions and the target microparticles. In this paper, the total distance that all microparticles are transported was selected as the goal function. An optimization approach based on the Kuhn-Munkres algorithm was applied to find the proper solution for target allocation. First, an adjacency matrix was established, where the estimated costs between each initial position of the microparticle and each goal position are the entries of the matrix. The estimated cost was calculated based on the Manhattan distance between the start position and the goal position, and the additional detour distance for obstacle avoidance was also taken into consideration, according to

(3) $$\begin{array}{c} E_{i-j}=D_{i-j}+\lambda \overset{m}{\underset{k=1}{\sum }}\left[\cos^{-1}\left(\frac{L_{k,i-j}}{R_{sys}}\right)R_{sys}-2\sqrt{{R_{sys}}^{2}-{L_{k,i-j}}^{2}}\right]+\overset{n-1}{\underset{w=1}{\sum }}\gamma_{w,i-j}R_{sys}\left(\pi -1\right), \end{array}$$

where *E*_*i*−*j*_ refers to the estimated cost from the start position of the *i*-th microparticle to the *j*-th goal position; *D*_*i*−*j*_ is the Manhattan distance between the start position of the *i*-th microparticle to *j*-th goal position; *λ* represents the influence factor of the extra distance for avoiding collisions with the obstacle on the estimated cost; *L*_*k*,*i*−*j*_ is the offset distance of the *k*-th obstacle relative to the straight line from the start position of the *i*-th microparticle to the *j*-th goal position; and *R*_*sys*_ is the estimated radius of the path for detour. *γ*_*w*,*i*−*j*_*R*_*sys*_(*π* − 1) refers to the estimated extra distance for the *i*-th target microparticle to avoid collision with the *j*-th target microparticle. *γ*_*w*,*i*−*j*_ is equal to 0 when the adjacency matrix is constructed for the first time.

The adjacency matrix was processed by the Hungarian method [40] and then transformed into a bipartite graph. The augmenting paths for the assignment were calculated iteratively until the maximum matching in bipartite graph was found. Then, the data in the adjacency matrix were updated: when the estimated cost for one microparticle is considered, the default goal positions of other target microparticles follow the latest matching scheme; *γ*_*w*,*i*−*j*_ in Equation (3) is no longer all equal to 0 but is determined by two line segments (one connects the start position of the *i*-th microparticle and the *j*-th goal position, and the other connects the *k*-th microparticle and its default goal position). The influencing factors include the angle between the two-line segments and the respective distances from the two starting positions (*i* and *j*) to the intersection of the two-line segments. The maximum matching process was repeated until the matching schemes of the latest two runs were consistent, which means that the goal assignment was completed.

#### 2.3.2. Determination of Optical Strategy

Typical path planning algorithms that depend on the models with fully known environmental information are unqualified for transporting microparticles at the microscale due to the existence of Brownian motion and the unpredictable responses of particles to the movement of the virtual electrode. Hence, a dynamic path planner based on the POMDP (partially observable Markov decision process) algorithm [41] was developed in this study, which takes randomness and unpredictability into account in the form of probability distribution matrixes. To further facilitate the computational effectiveness of the POMDP algorithm, a simplified method known as QMDP was utilized in this paper.

We transform the discretized transportation task of microparticles into a POMDP model. A 32 × 32 mesh divides the 2D workspace into multiple neighboring square units, and each unit represents a state of a spatial location. *S* = {*s*_1_, *s*_1_, *s*_1_, ⋯*s*_*n*_} refers to the set of all states in the workspace. The path planner splits the entire transportation task of an individual particle from the starting position to the goal position into a sequence of single-step motion decisions that transfer between the adjacent states. *A* = {*a*_1_, *a*_1_, *a*_1_, ⋯*a*_*n*_} refers to the set of all single-step actions, where the elements are vectors pointing to 8 adjacent square units and itself. *s* represents the current state of an individual particle, and *s*′ is the next state after the execution of action *a*. Each step of motion decisions formulated by the path planner essentially determines the optimal control policy *μ* from the set of actions *A* for all microparticles. Due to the randomness caused by Brownian motion and the inevitable error of observation, the position information of the obstacle is not completely determined and the response of the target to the virtual electrode cannot be completely predicted, so this uncertainty is modeled in the form of a probability distribution. The probability that a microparticle is currently in state *s* is denoted by *b*(*s*); *p*(*s*′|*s*, *a*) represents the probability of transferring from state *s* to a special state *s*′ after action *a*, where *b*(*s*) is calculated based on the observation probability distribution *O* = {*o*_1_, *o*_1_, *o*_1_, ⋯*o*_*n*_} and the sufficient statistics analysis of the control policy history.

Computing value functions *V*(*s*) is one of the most significant steps in a Markov decision process. QMDP improved the calculation efficiency by using the value function based on the estimate state in the MDP model to process the optimal decision making in the POMDP model. The value function of the state corresponding to the goal position is initially set to 100, while the value function of all possible states corresponding to the obstacle is set with the probability distribution *b*(*s*) as the weight and -50 as the base. The value functions of all states of the workspace are calculated iteratively until it finally converges, according to

(4) $$\begin{array}{c} V_{MDP}^{*}\left(s\right)=\underset{a}{\max}Q_{MDP}^{*}\left(s,a\right),\\ Q_{MDP}^{*}\left(s,a\right)=r\left(s,a\right)+\eta \underset{s^{\prime }}{\sum }V_{MDP}^{*}\left(s^{\prime }\right)p\left(\left.s^{\prime }\right|s,a\right), \end{array}$$

where *Q*_*MDP*_^∗^(*s*, *a*) represents the *Q* function for state *s* by executing action *a* in the MDP model, *η* is a discount rate, and *r*(*s*, *a*) refers to the immediate reward for state *s* by executing action *a*.

The probability distribution over the belief space *b*(*s*) is taken into consideration for calculating the optimal control policy *μ* of individual microparticles, which can be described as

(5) $$\begin{array}{c} u_{Q_{MDP}}\left(b\right)=\underset{a}{\arg\max}\left[\underset{s}{\sum }b\left(s\right)Q_{MDP}^{*}\left(s,a\right)\right]. \end{array}$$

#### 2.3.3. Priority Assignment

The setting of the different value functions for the target position and obstacle makes the path planner possess the ability to avoid collisions with free microparticles suspended in liquid. However, the conflict between the movement of multiple target microparticles is the more significant source of collision for the manipulation of massed targets in a limited space. Therefore, determining the optimal motion strategy for each microparticle separately is not satisfactory. Faced with potential conflicts between target microparticles, priority assignment methods are proposed.

Before every step of the motion decision-making, each microparticle is assigned two levels of priority: first-level priority and second-level priority. Microparticles of the same type have the same high-level priority, and the larger the outer diameter of the virtual electrode for different types of particles is, the higher the first-level priority is. Among the microparticles of the same type, the second-level priority is assigned in order according to the distance between the current position of the microparticle and the goal position from small to large. The second priorities of the microparticles are compared only when the first priorities are equal. In every step of the process of motion decision-making, the optimal strategies for microparticles are calculated in the order of priority. The next possible states of the microparticles with higher priority, with the optimal strategy as the action, will be assumed as states of obstacles to participate in the decision-making of microparticles with lower priority. Similarly, the next possible states of one microparticle with higher priority can be concluded as a set according to the probability distribution over the belief space. The motion decision after priority allocation is essentially to make microparticles with lower priority spend more transportation costs to avoid potential conflicts with higher-priority microparticles. Because the extra detour distance of microparticles that is farther from the goal positions or with a smaller radius of virtual electrode is usually smaller, such microparticles are assigned a lower priority.

### 2.4. Visual Feedback

**Figure 3:**
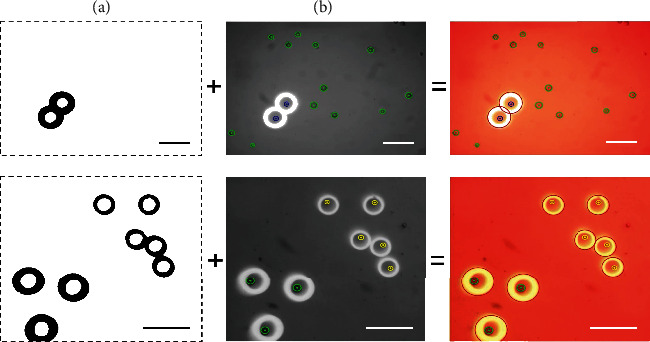
Differentiated image preprocessing and recognition of light patterns and microparticles. (a) Threshold method for recognition of light patterns. (b) Grayscale processing and Gaussian filter processing for recognition of microparticles. Scale bars, 100 *μ*m.

The visual feedback module provided real-time information of environment so that the path planner could determine every step of the strategy in time. In addition to the most basic position coordinates, the capabilities to differentiate target particles from nontarget particles and to distinguish the types of microparticles are essential for the path planner. To detect whether microparticles are at risk of escaping from the virtual electrode in real time, recognition of light patterns is also indispensable. In this paper, the near-circular edge features of light patterns and microparticles were detected by the Hough circle detection algorithm, but before that, differentiated image preprocessing is required according to the different characteristics of the microparticle and light pattern, as shown in Figure 3. For detailed information on image preprocessing, please refer to Materials and Methods 2 in the Supplementary Materials (available here). Microparticles are divided into different categories according to size by the *K*-means clustering algorithm. Nontarget microparticles were screened out by the Euclidean distance between the light pattern and microparticles. After detecting the position of the particles and light patterns in each frame, the trajectories of microparticles are tracked and predicted through the Kalman filter algorithm to avoid confusion of the microparticle's ID.

### 2.5. Cell Culture and Suspension

NIH/3T3 fibroblast cells were purchased from ATCC, USA, and maintained in a Petri dish with DMEM/F12 containing 10% fetal bovine serum (GIBCO, USA) and 1% penicillin-streptomycin (Solarbio, China). When the cell confluence area reached 80% in the Petri dish, cells were detached from the dish using trypsin (Solarbio, China). Cells were cultured under conditions of 37°C and 5% CO_2_ until the confluence area of cells was up to 80% in the Petri dish. Then, trypsin was used to separate the 3T3 cells from the substrate of the dish. After centrifugation and discarding the supernatant, the cell precipitate was diluted in deionized (DI) water supplemented with 5% glucose and 2% bovine serum albumin (Solarbio, China) before manipulation.

### 2.6. Statistical Analysis

All values were represented as mean ± standard deviation (SD). Two-way analysis of variance (ANOVA) with Student's *t*-test were used for data comparisons (^∗^*p* < 0.05 is considered statistically significant).

## 3. Results

### 3.1. Optimization of Virtual Electrode

**Figure 4:**
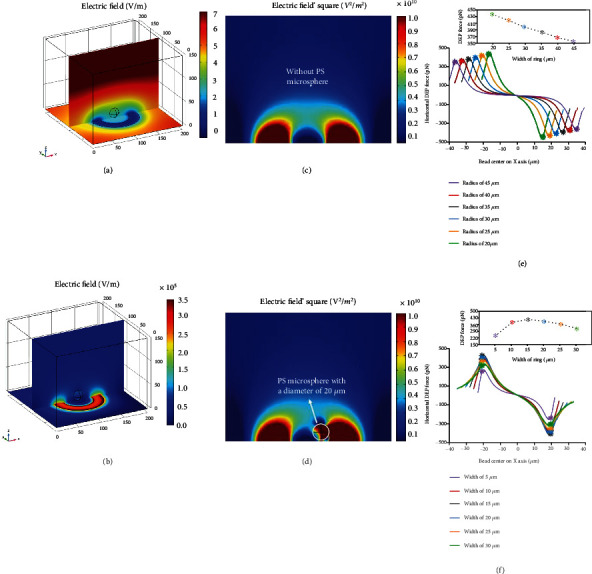
Simulation result by COMSOL Multiphysics. Distribution of (a) electronic potential and (b) electric field in an OET chip with a microparticle. Distribution of *E*^2^ in an OET chip (c) without microparticles and (d) with a microparticle positioned at the edge of the trap. Curves of the simulated DEP force along the *x*-axis for a microparticle provided by virtual electrodes (e) with the same width but different radius and (f) with the same radius but different width.

Based on the model in Section 2.2, the electric potential and field distribution of the OET chip with microparticle were simulated, as shown in Figures 4(a) and 4(b). Since the model is centrally symmetrical, only two slices are displayed as representatives to express the three-dimensional distribution of the electric field and potential of the whole OET chip. It is obvious that the existence of microparticles has a significant impact on the distribution and intensity of the electric field around the microparticle in Figure 4(b). As shown in Figures 4(c) and 4(d), the difference between the results of the |*E*|^2^ distribution obtained from the simulation model with or without microsphere is not negligible. The inconsistency in electrical properties (permittivity and conductivity) of the microparticles and the suspended liquid as well as the induced dipole moment of the dielectric particles excited by the external electric field are the reasons for the change of the local electric field inside and around the microparticle. For the detailed analysis about the various impacts of the microparticle of different size and materials on the changes of the electric field, please refer to Materials and Methods 1 in the Supplementary Materials. Since the accuracy of ∇|*E*|^2^ is critical for estimation of the dielectrophoretic force, it is essential that the existence of the microsphere be considered in the simulation model. The method of Equation (1) could qualitatively determine the direction of the DEP force but does not have the ability to accurately estimate the magnitude of the dielectrophoretic force. Therefore, the Maxwell stress tensor method (Equation (2)) is used for approximating the DEP force rather than the dipole approximation method. We traverse the *x*-axis coordinates of the microspheres from one end of the OET trap to the other for obtaining the DEP force distribution in one dimension.

For transportation of microparticles, one of the most important factors that determines the efficiency is the maximum movement speed, which is determined by the DEP force provided by the virtual electrode. Therefore, we estimated the magnitude of the dielectrophoretic force under the virtual electrode of different radius (radius of inner circle) and width (the radius of the outer circle minus the radius of the inner circle) conditions to obtain the appropriate size of the virtual electrode. As shown in Figures 4(e) and 4(f), the closer the microparticle is to the inner circle of the virtual electrode, the greater the DEP force exerted on the microparticle. The DEP force at the inner circle of the virtual electrode is represented by ^∗^ with different colors. Figure 4(e) shows the DEP force exerted on the microparticle by five virtual electrodes with the same width (20 *μ*m) and different radii (20 *μ*m, 25 *μ*m, 30 *μ*m, 35 *μ*m, 40 *μ*m, and 45 *μ*m). When the width of the virtual electrode does not change, the force at the inner circle increases slightly as the radius decreases. A too small radius will cause microparticles to effortlessly escape from the OET trap, so we choose a radius of 20 *μ*m for the next step of the simulation. As shown in Figure 4(f), the horizontal DEP force at the inner circle is not positively correlated with the width of the virtual electrode but reaches its maximum value at 15 *μ*m in width. The possible reason is that when the size of the virtual electrode is too large compared to the central nonilluminated area, the gradient of the electric field distribution in the local region will decrease, which leads to a lower total amount of ∇|*E*|^2^ inside the microsphere. The simulation results demonstrate that a virtual electrode with a larger size or smaller size does not produce a greater horizontal DEP force. Appropriate reference values of the radius and width for the virtual electrode corresponding to microparticles of different sizes and materials could be obtained through this simulation method.

### 3.2. Particle Transportation with Real-Time Path Planner

**Figure 5:**
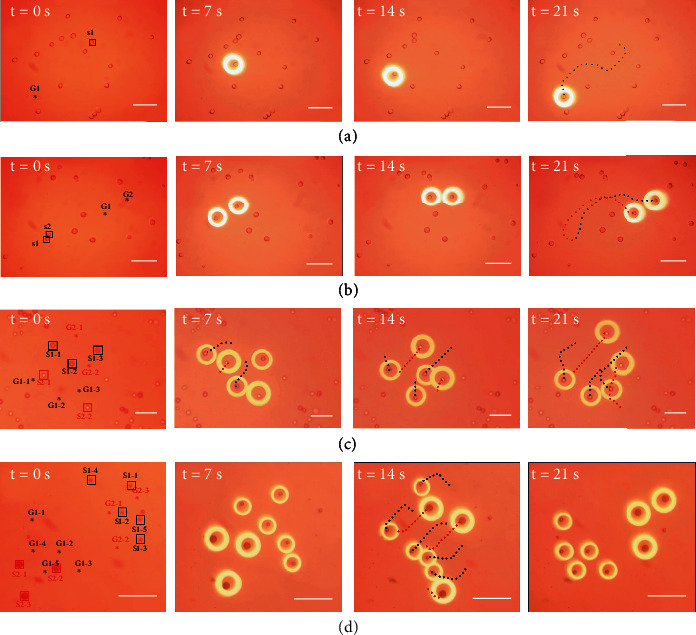
Experiments of transporting target microparticles to designated positions. (a) A 3T3 cell was transported to the goal position by avoiding collision with obstacles. (b) Two 3T3 cells with close start positions successfully avoided collisions with each other and were transported to the corresponding goal positions. (c) Two 3T3 cells and three polystyrene particles were transported to the goal positions simultaneously. (d) Two groups of polystyrene particles of different sizes were transported in the direction pointing to each other's starting position and successfully rendezvous. Scale bars, 100 *μ*m.

NIH/3T3 cells and two different sizes of polystyrene microspheres (10 *μ*m and 20 *μ*m) were swimmingly transported to the goal position under automation control as shown in Figure 5. In Figure 5, the upper left corner of the image represents time in this current state. *S*_*i*−*j*_ and *G*_*i*−*j*_, respectively, represent the start position and goal position of the *j*-th particle belonging to the *i*-th type (different color for distinguishing the type of particles). The dashed lines formed by discrete points are to demonstrate all or part of the particle's trajectories.

A 3T3 cell in Figure 5(a) was transported to the goal position approximately 360 *μ*m away in a relatively crowded environment (containing 16 obstacles). The path planner prompted the 3T3 cell to determine the direction with fewer obstacles and circumvent collision with a small-time cost. Figure 5(b) demonstrates the effectiveness of the path planning algorithm in circumventing collisions between trapped microparticles. The two target microparticles were so close at the starting position (*S*1 and *S*2) that they were about to stick together. Therefore, they were first transported in the direction of increasing distance between each other and closer to the goal positions until the relative position is safe enough. Note that the circumstance where the distance between trapped microparticles is lower than the safety threshold only appeared near start positions and goal positions but not in the main part of the trajectory because of the efforts of the path planner to avoid potential collisions during the transportation of particles. In Figure 5(c), two 3T3 cells and three polystyrene microparticles were trapped by virtual electrodes with different geometric parameters and were finally transported to corresponding goal positions, which verify the ability of the proposed manipulation methods for transporting microparticles with different materials. We then designed an extreme experiment to verify the capability of the path planner for collision avoidance in manipulation tasks with different types of particles (Figure 5(d)). The two microparticle swarms of different sizes (10 *μ*m and 20 *μ*m) were located in the lower left corner and upper right corner of the sight view at the initial moment, while the target positions were artificially inverted, which resulted in the motion of two types of microparticles being almost opposite. During the rendezvous of two particle swarms, the potential risk of collision greatly increased. Rendezvous and separation of particles were accomplished by staggered advancing with a small circumvention cost and arranged in a triangular and trapezoidal distribution pattern at goal positions. *G*1 − 1 is closer to *S*1 − 4 rather than *G*1 − 4, but the path planner assigned *G*1 − 4 as the goal position of *S*1 − 4 because the expectation of goal assignment is to achieve the global optimal solution.

### 3.3. Micropatterning of Multiple Particles

**Figure 6:**
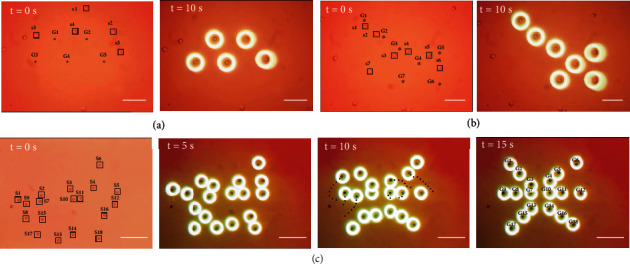
Experiments of applying discretized manipulation method for micropatterning. (a) A “trapezoid” formed by transporting 5 3T3 cells within 10 s. (b) A “arrow” pointing to the lower right formed by transporting 7 3T3 cells within 10 s. (c) A radial pattern that mimics the liver lobules by transporting 18 polystyrene microspheres within 21 s. Scale bars, 100 *μ*m.

Simultaneous transportation of particles is widely used in many micromanipulation tasks in biomedical studies, including micropatterning. In addition to being part of the construction of artificial cellular models with biological tissue-mimicking architecture, the results of biological micropatterning are also experimental platforms for drug testing, cell culture, and cell behavior research. As mentioned in Section 1, preliminary experiments of applying the discretized manipulation method for micropatterning were carried out. The “trapezoids” and “arrow” containing 3T3 cells were arranged by the path planner within 10 seconds, as shown in Figures 6(a) and 6(b). It is worth noting that it took only 15 seconds for the 18 polystyrene microspheres to be transported to the target positions and form a radial pattern that mimics the liver lobules (Figure 6(c)). The result demonstrates the efficiency of the path planner and the scalability for the number of target particles, which benefits from the modules of goal assignment and dynamic obstacle avoidance. Part of the trajectory of some microparticles is marked with dashed lines to visually illustrate the effort to avoid collisions.

## 4. Discussion

In this paper, we proposed a discretized manipulation method for transporting multiple types of microparticles with an optoelectronic tweezer platform. The influence of geometric parameters of the “virtual electrode” on the manipulation of dielectric microparticles based on the ODEP mechanism is simulated and discussed to investigate the appropriate size of light patterns corresponding to each type of microparticles. On this basis, the simultaneous transport experiments of microparticles of different sizes or materials were performed to verify the adaptability of the mentioned method to various targets. To improve the efficiency of the multiparticle transport as much as possible, an improved goal assignment method based on the Hungarian method is developed by taking the additional detour distance into account. In terms of collision avoidance with potential obstacles, the centralized path planner based on the POMDP algorithm was proposed for synchronized path planning of all microtargets including cells. A two-level priority system was established to revise the motion decision for avoiding potential collision of multiple target microparticles. The experiments of transporting multiple microparticles (including cells) to corresponding goal positions in relatively crowded liquid environments demonstrated the effectiveness and reliability of the proposed path planner. In addition, experiments of transporting cells to designated positions in specific patterns are performed as a preliminary attempt of cell patterning, which indicated the feasibility and potential of applying the mentioned methods to biomedical studies. The experiment results demonstrate that the proposed method is able to sufficiently utilize the versatility and high throughput of optoelectronic tweezers, which provide a novel solution for massively parallel manipulation of microtargets.

In the future, the proposed method is expected to be utilized for more intricate biomedical experiments with larger-scale cells, e.g., construction of cellular modules with more physiological functions, optimization of culture conditions, and studies of cell-cell interactions. However, there are still some technical challenges that require more attention. For the significant decrease in computational efficiency of the path planner with the increased number of targets for the transportation task, the method of computing in batches (e.g., Convolutional Neural Network) may be potential solutions. To maintain the cellular micropatterns after transportation of cells, it is critical to develop the feasible schemes for in situ encapsulation of cells or cell-adherent culture in OET chips. In addition, the position accuracy of the microsphere during movement needs to be further improved without a closed-loop motion controller. Therefore, we will further explore and analyze the dynamics and kinematics of microparticles actuated by optoelectronic tweezers, as well as the interaction mechanism between virtual electrodes and microspheres.

## Data Availability

The data are freely available upon request.

## Supplementary Materials

Supplementary Materials Supplementary Materials and Method 1: various impacts of the existence of microparticle with different sizes and materials on the electric field. Supplementary Materials and Method 2: image preprocessing for recognition of light patterns and microparticles.
