# Supplementary material for: POMDP-Based Real-Time Path Planning for Manipulation of Multiple Microparticles via Optoelectronic Tweezers
Source: Cyborg Bionic Syst. 2022 Nov 2;2022:9890607. doi: 10.34133/2022/9890607 (PMC9652702; doi:10.34133/2022/9890607)
Supplement: Supplementary Materials — and Method 1: various impacts of the existence of microparticle with different sizes and materials on the electric field. Supplementary Materials and Method 2: image preprocessing for recognition of light patterns and microparticles. [file 9890607.f1.docx]

POMDP-Based Real-Time Path Planning for Manipulation of Multiple Microparticles via Optoelectronic Tweezers

Jiaxin Liu,^2^ Huaping Wang,^1^* Menghua Liu,^2^ Ran Zhao,^2^ Yanfeng Zhao,^2^ Tao Sun,^3^ Qing Shi^4^

Supplementary Materials

Affiliations

1. Key Laboratory of Biomimetic Robots and Systems (Beijing Institute of Technology), Ministry of Education, Beijing 100081, China.
2. Intelligent Robotics Institute, School of Mechatronical Engineering, Beijing Institute of Technology, Beijing 100081, China.
3. School of Medical Technology, Beijing Institute of Technology, Beijing 100081, China.
4. Beijing Advanced Innovation Center for Intelligent Robots and Systems, Beijing Institute of Technology, Beijing 100081, China.

* Corresponding author. Email: wanghuaping@bit.edu.cn (H.W.)

**1. Impacts of the existence of microparticle with different sizes and materials on the electric field.**

Add contents in Supplementary Materials: “We explore the impact of the existence of particles with different sizes and materials on the local electric field around the microparticle through a series of simulations with different parameter settings. The shape of light pattern in the simulation model is a ring. Since the position of the microparticles is not in the center of the light pattern during the movement, the microspheres are placed off-center in the simulation model to be consistent with the actual situation. The simulation results are shown as the distribution of electric field intensity in a specific cut plane (which is perpendicular to the horizontal plane and through the center of the light pattern and the center of the particle) in Figure S1 and S2.

In the optically-induced non-uniform electric field, it has been widely reported that the main material properties relevant to the dielectrophoretic force exerted on particles are permittivity and conductivity. Figure S1 shows the differential impact of the existence of particles with different conductivity and permittivity on the electric field intensity. $\varepsilon_{1}$ and $\sigma_{1}$ represent the permittivity and conductivity of the liquid, respectively. Figure S1 (A) and (C) shows the distribution of electric field intensity when the particles have the same permittivity ($\varepsilon_{1}$) but different conductivities (0.25$\sigma_{1}$, 0.5$\sigma_{1}$, 0.5$\sigma_{1}$, 2$\sigma_{1}$, and 4$\sigma_{1}$ respectively). While the effect of the particle’s permittivity on the distribution of electric field intensity is shown in Fig. S1 (B) and (D). It is obvious that the impact of the existence of the microparticle on the electric field is more significant when the difference in electrical properties of the microparticle and the suspended liquid is larger. In addition, the simulations under different frequency present how strongly conductivity and permittivity of microparticles affect the intensity of the local electric field with respect to frequency. When the frequency is relatively large (e.g., 50$MHz$for Figure S1 (A) and (B)), the existence of microparticles with different permittivity causes large variations in the distribution of electric field intensity. While the change of conductivity has little effect on the electric field. When the frequency is relatively small (e.g., 50$KHz$for Figure S1 (C) and (D)), conductivity rather than permittivity of microparticles dominates the effect on the electric field intensity. In this study, the impact of the existence of microparticles with different materials on the electric field is basically divided into two categories, which depend on the conductivity and permittivity of the microparticles compared to the suspended liquid. When the conductivity or permittivity of the particle is higher than that of the liquid, the electric field intensity inside the microsphere becomes larger and the electric field intensity of the local electric field around becomes smaller. While the former becomes smaller and the latter becomes larger when the conductivity or permittivity of the particle is lower than that of the liquid.


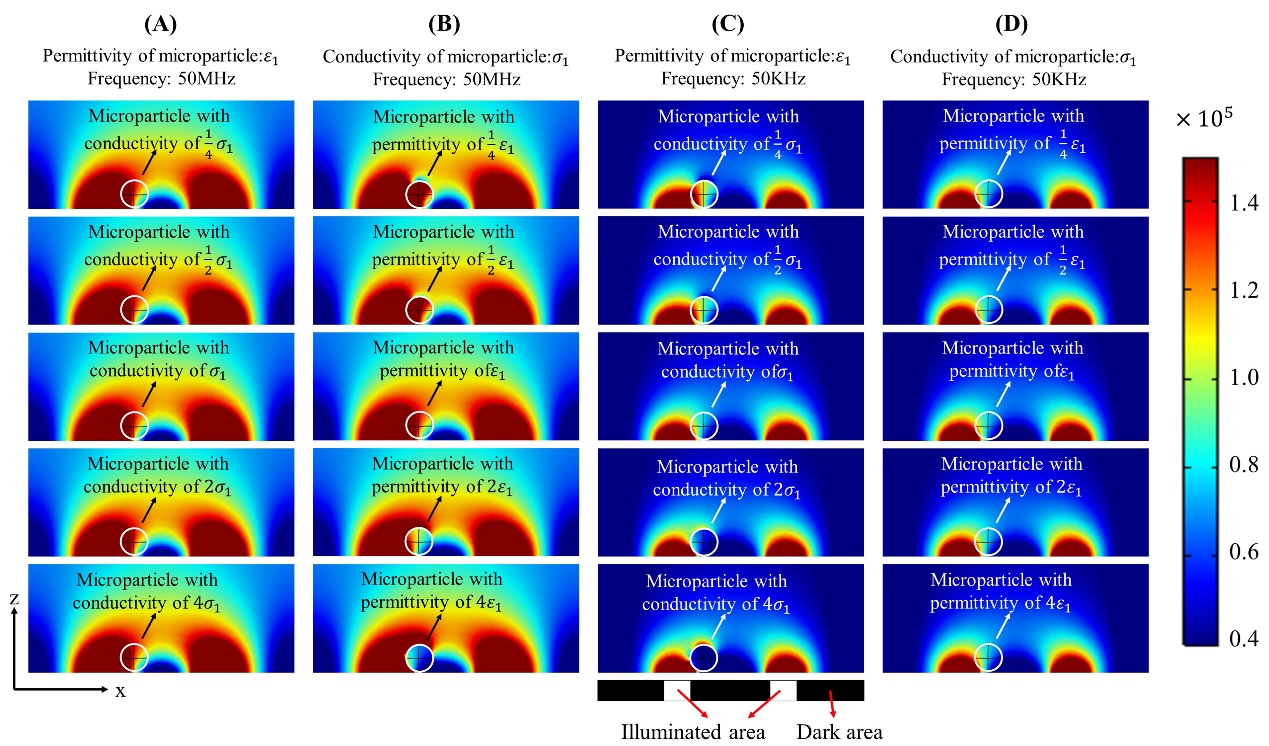


**Figure S1**. Various impacts of the existence of microparticles with different materials on the intensity of the electric field. Simulation results at 50$MHz$ with the existence of the microparticle of the same permittivity (A) and the same conductivity (B). Simulation results at 50$KHz$ with the existence of microparticles of the same permittivity (C) and the same conductivity (D).

In addition to materials, the size of the microparticles is another significant factor in the effect on the electric field. Figures S2 (A) illustrates the electric field intensity distribution with the existence of polystyrene microsphere (solder beads for Figures S2 (B)) of different sizes. For microparticles of the same material, a larger particle size makes the magnitude of impact on the electric field intensity greater but does not change the category of impact.


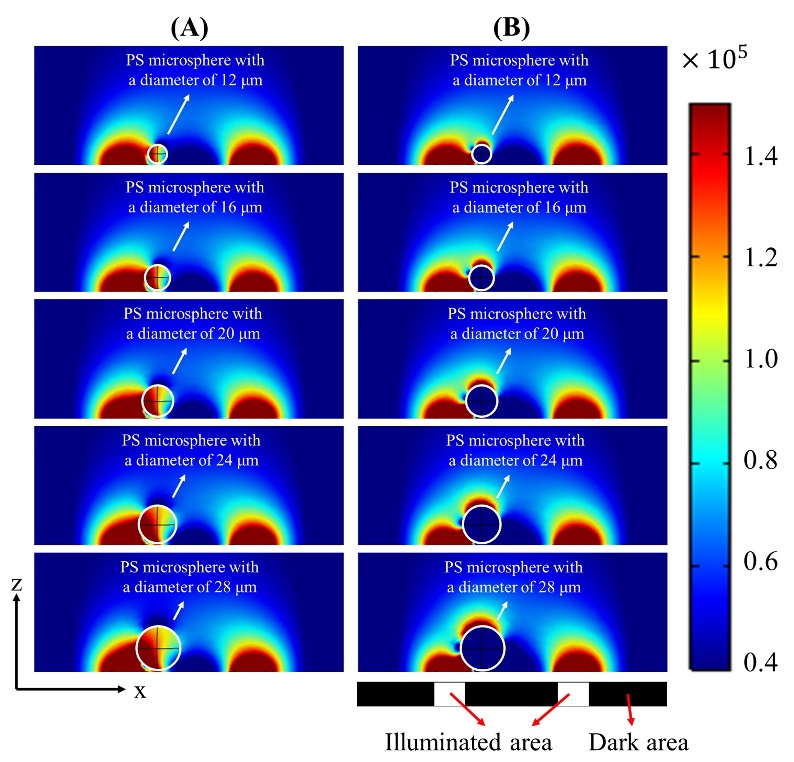


**Figure** **S2**. Various impacts of the existence of microparticles (polystyrene microspheres for (A) and solder beads for (B)) with different sizes on the intensity of the electric field.

**2. Image preprocessing for recognition** **of light patterns and microparticles.**

Different methods of image preprocessing are required for the recognition of microparticles and light patterns according to their differentiated features in the image. The high-frequency characteristics of the light pattern in the image are not significant due to the scattering effect of light in the liquid environment and the existence of a filter in front of the CCD camera. For recognition of light patterns, the image captured by CCD was converted into a binary image: black circles (light pattern) on a white background. In order to lessen the interference caused by changes in image brightness to feature recognition, the Otsu algorithm was applied to determine the threshold for binarization, which can be described by:

$$g=\max_{i=0}^{255} (w_{i,0}\times(u_{i,0}-u_{2})^{2}+w_{i,1}\times(u_{i,1}-u_{2})^{2})$$

where $i$ represent the gray value of the iteration, $w_{i,0}$ and $w_{i,1}$ are the proportion of pixels belonging to the foreground (gray level of 0 ∼ $i$) and background (gray level of $i$ ∼ 255) in the entire image, respectively. $u_{i,0}$, $u_{i,1}$ and $u_{2}$ refer to the average gray level of the foreground, background, and whole image, respectively.

The microbeads and floating cells are spherical and have obvious circular features in the two-dimensional image. Since there is no obvious color difference between target microparticles and obstacles to be detected, the three-channel RGB image was converted into a single-channel grayscale image to shorten the detection and recognition time. Then the grayscale image was filtered to eliminate the noise generated in the process of imaging and data transmission with a 5×5 Gaussian filter operator, where $G\left(u,v\right)$ is the Gaussian filter operator:

$$I_{fianal}\left(x,y\right)=\left(I_{red}\times0.299+I_{green}\times0.587+I_{blue}\times0.114\right)\times G\left(u,v\right)$$

The preprocessed image was then imported into the Hough circle detection module.
